# Editorial to “Pre‐procedural imaging guiding ventricular tachycardia ablation in structural heart disease”

**DOI:** 10.1002/joa3.13211

**Published:** 2025-01-14

**Authors:** Yoshiaki Mizutani, Satoshi Yanagisawa, Yasuya Inden

**Affiliations:** ^1^ Department of Cardiology Yokkaichi Municipal Hospital Yokkaichi Mie Japan; ^2^ Department of Cardiology Nagoya University Graduate School of Medicine Nagoya Aichi Japan

Ventricular tachycardia (VT) often occurs in patients with damaged hearts and decreased cardiac function, such as those with ischemic cardiomyopathy (ICM). Defibrillation therapy with an implantable cardioverter‐defibrillator (ICD) improves prognosis in these patients for both primary and secondary prevention. However, characteristics of nonischemic cardiomyopathy (NICM) are different from those of ICM, leading to variability in prognoses following ICD implantation, especially for primary prevention, and presenting challenges in VT management through catheter ablation. Given the increasing global prevalence of NICM and recent advancements in catheter ablation techniques and imaging modalities, improved prognoses and effective approaches for catheter ablation in patients with NICM are expected.

In this issue of the *Journal of arrhythmia*, Ferreira et al.^1^ evaluated the safety and efficacy of VT ablation in patients with NICM and ICM using the ADAS 3D system (ADAS3D Medical, Barcelona, Spain). A total of 102 patients with VT were included in this study (ICM, 75 patients; NICM, 27 patients). Multidetector computed tomography (MDCT), and late gadolinium enhancement cardiac magnetic resonance (LGE‐CMR) were used for preprocedural imaging. These were integrated into mapping systems and segmented using ADAS 3D software. The key points of this study are as follows: First, procedural data revealed no significant differences in VT inducibility between the ICM and NICM groups. Approximately half of the patients in each group no longer exhibited VT inducibility, possibly because of the elimination of all late potentials, achieved through preprocedural imaging complemented with the ADAS 3D system and its integration into the three‐dimensional electroanatomical mapping system. Second, cumulative survival free from appropriate ICD shocks was similar between the ICM and NICM groups. This suggests that preprocedural imaging‐guided ablation for VT may be equally beneficial in patients with NICM and as it is in patients with ICM. Much of the past randomized studies for evaluating VT ablation have been conducted in patients with ICM, while large‐scale prospective randomized studies for patients with NICM remain lacking.^2^, ^3^ Previous studies have demonstrated inferior outcomes following VT ablation in patients with NICM compared to those with ICM, possibly because of the heterogenous VT substrate in patients with NICM.^4^ Typically, the substrate of NICM is characterized by an increased prevalence of damaged tissue expanding into intramyocardial and epicardial sites, which is higher than that of ICM. This complexity poses challenges, such as reduced catheter accessibility and insufficient thermal energy delivery to deep myocardial layers, resulting in a lower VT termination rates and poorer procedural outcomes.^5^ This result aligns with the findings of current study, with >70% of patients with NICM requiring epicardial ablation compared with >90% of patients with ICM undergoing endocardial ablation.^1^ Similar success rates and outcomes observed between the patients with ICM and NICM, in this study, may lead to a better perspective of the ablation approach in these high‐risk populations with poor prognoses. In addition, recent advances in technology, updated ablation devices, and imaging solutions might be associated with increased success rates and favorable outcomes. However, caution is warranted when interpreting these results because of the study's nonrandomized design and significant differences in baseline characteristics between the two etiology groups. The relevant differences in mean age, prevalence of chronic kidney disease, and electrical storm may have substantially affected the procedural outcomes and success rates of ablations.^1^

Furthermore, the current study assessed the endpoint of VT episodes treated with ICD shocks but did not account for episodes managed with anti‐tachycardia pacing (ATP) following the procedure. Although ATP therapy effectively reduces the need for shocks and mitigates anxiety in patients, its use is associated with poorer prognosis and carries the risk of accelerating VT cycle length and subsequent ICD shock treatment. Thus, additional analysis including patients with ATP‐activated or nonsustained VT, as well as ATP‐induced fast VT/VF resulting in shock activation, would further strengthen this study. Moreover, the sensitivity and specificity of preprocedural imaging for identifying local abnormal ventricular activities during invasive mapping were suboptimal. MDCT demonstrated 66.7% sensitivity and 60.0% specificity, whereas MRI exhibited 57.1% sensitivity and 74.6% specificity. However, in cases where MDCT and MRI were inaccurate, the region of interest were adjacent to the predicted cardiac segment. This underscores the utility of preprocedural imaging protocols in targeting areas near the VT circuit or a short distance away. Focusing on the area of interest and their vicinity may help reduce treatment time and minimize excessive energy delivery.

The integration of advanced preprocedural imaging modalities, such as the ADAS 3D system, is useful in the identification and localization of arrhythmogenic substrates, even in deeper layers of the myocardium. However, challenges remain in addressing critical isthmuses where standard thermal ablation heating using high‐power energy cannot reach, possibly owing to the long anatomical distance or risk of complications to adjacent tissue. Further systematic evaluations of preoperative imaging protocols and novel ablation modalities (e.g., pulsed‐field ablation) for VT ablation using large‐scale samples are required.

## CONFLICT OF INTEREST STATEMENT

The authors declare no conflicts of interest.
